# Diagnosis of follicular thyroid cancer based on the presence of a metastasis in the vertebral column – case report

**DOI:** 10.1186/1756-6614-6-S2-A32

**Published:** 2013-04-05

**Authors:** Monika Koziołek, M Wolska, A Sieradzka, M Jakuszewski, K Karpińska-Kaczmarczyk, M Bilski, Anhelli Syrenicz

**Affiliations:** 1 Department of Endocrinology, Metabolic Diseases and Internal Diseases, Pomeranian Medical University in Szczecin, Szczecin, Poland; 2 Clinic of Orthopedy and Traumatology, Pomeranian Medical University in Szczecin, Szczecin, Poland; 3 Department of Pathomorphology, Pomeranian Medical University in Szczecin, Szczecin, Poland; 4 Department of Endocrinology and Radioisotope Therapy, Military Institute of Medicine, Szczecin, Poland

## Introduction

Follicular thyroid cancer, similar to follicular adenoma, usually takes the form of a round, encapsulated tumour. Cytological examination does not allow a firm diagnosis of follicular thyroid cancer to be made. Follicular thyroid cancer can be diagnosed by histopathological examination only after demonstrating the infiltration of the capsule by the tumour and/or vessel invasion. The tumour spreads mainly through the bloodstream causing metastasis to the lungs and bones. We present a case of a 70-year-old woman who has been diagnosed with a follicular thyroid carcinoma based on the presence of a metastasis to the bones.

## Case report

A 70-year-old woman who had undergone a subtotal thyroidectomy in 2007 because of a nodular goiter, diagnosed as struma nodosa on the histopathological examination, was admitted to the Clinic of Orthopedy and Traumatology with a pathological fracture of the first lumbar vertebra (10.2012). The spine MRI scan revealed a 30mm pathological mass in the L1 vertebral body; therefore a removal of the L1 vertebral body was performed, followed by a histopathological examination. The result revealed metastasis carcinomatosa, carcinoma folliculae glandulae thyroideae. The patient was directed to the Department of Surgery, where a total thyroidectomy with the removal of lymph nodes was performed (12.2012). Based on the histopathological result, the patient was diagnosed with struma macro et microfollicularis and reactive lymph nodes. The histopathological result of the material from the corpus vertebrae was verified, and the previous diagnosis of follicular cancer was confirmed. Accordingly, specimens from the thyroid gland obtained during the first thyroidectomy in 2007 were verified, yielding the following result: encapsulated follicular thyroid cancer with infiltration of the capsule; infiltration of the vessels was not demonstrated - minimally invasive follicular carcinoma. 18F-Fluoride PET/CT was performed which revealed metastatic metabolically active lesion in L1 vertebral body. The patient will be treated with radioactive iodine.

## Conclusions

Diagnosis of follicular thyroid cancer, especially its minimally invasive forms, may pose difficulty even when based on post-surgical histopathological specimens.

